# Supplementary material for: Lower Extremity Amputation and Peripheral Revascularisation Rates in Romania and Their Relationship with Comorbidities and Vascular Care
Source: J Clin Med. 2023 Dec 21;13(1):52. doi: 10.3390/jcm13010052 (PMC10779533; doi:10.3390/jcm13010052)
Supplement: Supplementary file 1 [file jcm-13-00052-s001.zip › jcm-2705854-supplementary.pdf]

Table S1: RO-DRG v1. procedure codes

No.	Code	Procedure
**Revascularization procedures**		
1	H15103	Percutaneous transluminal balloon angioplasty with stenting, single stent
2	H15104	Percutaneous transluminal balloon angioplasty with stenting, multiple stents
3	H15101	Percutaneous transluminal balloon angioplasty
4	H10013	Embolectomy or thrombectomy of femoral artery, unilateral
5	H10014	Embolectomy or thrombectomy of popliteal artery, unilateral
6	H10502	Patch graft of artery using synthetic material
7	H11021	Femoro-popliteal bypass using synthetic material, above knee anastomosis, unilateral
8	H10012	Embolectomy or thrombectomy of iliac artery, unilateral
9	H10015	Embolectomy or thrombectomy of tibial artery, unilateral
10	H10501	Patch graft of artery using autologous material
11	H10102	Embolectomy or thrombectomy of bypass graft of artery of extremities
12	H12801	Procurement of vein from limb for bypass or replacement graft, unilateral
13	H10016	Embolectomy or thrombectomy of other artery
14	H11009	Aorto-femoro-femoral bypass using synthetic material
15	H11022	Femoro-popliteal bypass using synthetic material, below knee anastomosis, unilateral
16	H11012	Iliofemoral bypass using synthetic material
17	H10911	Femoro-popliteal bypass using vein, above knee anastomosis
18	H09901	Endarterectomy in conjunction with arterial bypass to prepare site for anastomosis
19	H11008	Aorto-femoral bypass using synthetic material
20	H15004	Excision of bypass graft from limb
21	H10912	Femoro-popliteal bypass using vein, below knee anastomosis
22	H11025	Other arterial bypass graft using synthetic material
23	H10915	Other arterial bypass using vein
24	H11102	Femoro-femoral crossover bypass
25	H10913	Femoral to proximal tibial or peroneal artery bypass using vein
26	H10101	Embolectomy or thrombectomy of bypass graft of artery of trunk
27	H10922	Popliteal-tibial bypass using vein
28	H11010	Aorto-iliac bypass using synthetic material
29	H15106	Open transluminal balloon angioplasty with stenting, multiple stents
30	H11015	Axillo-femoral bypass using synthetic material
31	H15102	Open transluminal balloon angioplasty
32	H10914	Femoral to distal tibial or peroneal artery bypass using vein
33	H11016	Axillo-femoro-femoral bypass using synthetic material
34	H11011	Aorto-ilio-femoral bypass using synthetic material
35	H11101	Iliofemoral crossover bypass
36	H11105	Femoral to tibial or peroneal artery bypass using composite graft, unilateral
37	H15201	Percutaneous peripheral laser angioplasty

38	H10906	Iliofemoral bypass using vein
39	H15105	Open transluminal balloon angioplasty with stenting, single stent
40	H11023	Femoral to proximal tibial or peroneal artery bypass using synthetic material, unilateral
41	H11107	Femoral artery sequential bypass using synthetic material, unilateral
42	H11034	Aorto-femoro-popliteal bypass using synthetic material
43	H11024	Femoral to distal tibial or peroneal artery bypass using synthetic material, unilateral
44	H11106	Femoral artery sequential bypass using vein, unilateral
45	H11036	Popliteal-tibial bypass using synthetic material
46	H11104	Femoro-popliteal bypass using composite graft, below knee anastomosis, unilateral
47	H15202	Open peripheral laser angioplasty
48	H10920	Aorto-femoro-popliteal bypass using vein
49	H11014	Subclavian-femoro-femoral bypass using synthetic material
50	H11035	Ilio-iliac bypass using synthetic material
51	H11103	Femoro-popliteal bypass using composite graft, above knee anastomosis, unilateral
52	H13304	Sapheno-femoral vein cross leg bypass
53	H15003	Excision of axillo-femoral or femoro-femoral bypass graft
54	H10921	Ilio-iliac bypass using vein
55	H11013	Subclavian-femoral bypass using synthetic material
56	H11026	Aorto-subclavian bypass using synthetic material
57	H13303	Sapheno-iliac vein cross leg bypass
Amputation procedures		
58	O11601	Amputation above knee
59	O16301	Amputation of toe, unilateral
60	O16302	Amputation of toe including metatarsal bone, unilateral
61	O16306	Transmetatarsal amputation, unilateral
62	O13603	Amputation below knee
63	O19307	Reamputation of amputation stump, unilateral
64	O16305	Midtarsal amputation, unilateral
65	O11602	Amputation at hip
66	O16304	Amputation of ankle through malleoli of tibia and fibula, unilateral

Table S2. –ICD-10-AM3 diagnosis codes

**No.**	**ICD-10-AM3 code**	**Diagnosis**
Risk factors		
1	E09.01	Glucose intolerance regulation with peripheric angiopathy without gangrene
2	E09.02	Glucose intolerance regulation with peripheric angiopathy with gangrene
3	E10.11	Type 1 diabetes mellitus with acidocetosis, without coma
4	E10.12	Type 1 diabetes mellitus with acidocetosis, with coma
5	E10.13	Type 1 diabetes mellitus with lactic acidosis, without coma
6	E10.14	Type 1 diabetes mellitus with lactic acidosis, with coma
7	E10.15	Type 1 diabetes mellitus with ketoacidosis, with lactic acidosis, without coma

8	E10.16	Type 1 diabetes mellitus with ketoacidosis, with lactic acidosis, with coma
9	E10.21	Type 1 diabetes mellitus with incipient diabetic nephropathy
10	E10.22	Type 1 diabetes mellitus with established or advanced kidney disease
11	E10.23	Type 1 diabetes mellitus with end stage renal disease
12	E10.29	Type 1 diabetes mellitus with other specified kidney complication
13	E10.31	Type 1 diabetes mellitus with background retinopathy
14	E10.32	Type 1 diabetes mellitus with preproliferative retinopathy
15	E10.33	Type 1 diabetes mellitus with proliferative retinopathy
16	E10.34	Type 1 diabetes mellitus with other retinopathy
17	E10.35	Type 1 diabetes mellitus with advanced ophthalmic disease
18	E10.36	Type 1 diabetes mellitus with diabetic cataract
19	E10.39	Type 1 diabetes mellitus with other specified ophthalmic complication
20	E10.40	Type 1 diabetes mellitus with unspecified neuropathy
21	E10.41	Type 1 diabetes mellitus with mononeuropathy
22	E10.42	Type 1 diabetes mellitus with polyneuropathy
23	E10.43	Type 1 diabetes mellitus with autonomic neuropathy
24	E10.49	Type 1 diabetes mellitus with specified neurologic complications
25	E10.51	Type 1 diabetes mellitus with peripheral angiopathy, without gangrene
26	E10.52	Type 1 diabetes mellitus with peripheral angiopathy with gangrene
27	E10.53	Type 1 diabetes mellitus with ischemic diabetic cardiopathy
28	E10.61	Type 1 diabetes mellitus with musculoskeletal and connective tissue complication
29	E10.62	Type 1 diabetes mellitus with skin and subcutaneous tissue complication
30	E10.63	Type 1 diabetes mellitus with periodontal complication
31	E10.64	Type 1 diabetes mellitus with hypoglycaemia
32	E10.65	Type 1 diabetes mellitus with poor control
33	E10.69	Type 1 diabetes mellitus with other specified complication
34	E10.71	Type 1 diabetes mellitus with multiple microvascular complications
35	E10.73	Type 1 diabetes mellitus with foot ulcer, multiple causes
36	E10.8	Type 1 diabetes mellitus with unspecified complications
37	E10.9	Type 1 diabetes mellitus without complications
38	E11.01	Type 2 diabetes mellitus with hyperosmolarity without noncetotic hiperglicaemic-hiperosmotic coma
39	E11.02	Type 2 diabetes mellitus with hyperosmotic coma
40	E11.11	Type 2 diabetes mellitus with ketoacidosis, without coma
41	E11.12	Type 2 diabetes mellitus with ketoacidosis, with coma
42	E11.13	Type 2 diabetes mellitus with lactic acidosis, without coma
43	E11.14	Type 2 diabetes mellitus with lactic acidosis, with coma
44	E11.15	Type 2 diabetes mellitus with ketoacidosis, with lactic acidosis, without coma
45	E11.16	Type 2 diabetes mellitus with ketoacidosis, with lactic acidosis, with coma
46	E11.21	Type 2 diabetes mellitus with incipient diabetic nephropathy
47	E11.22	Type 2 diabetes mellitus with established or advanced kidney disease

48	E11.23	Type 2 diabetes mellitus with end stage renal disease
49	E11.29	Type 2 diabetes mellitus with other specified kidney complication
50	E11.31	Type 2 diabetes mellitus with background retinopathy
51	E11.32	Type 2 diabetes mellitus with preproliferative retinopathy
52	E11.33	Type 2 diabetes mellitus with proliferative retinopathy
53	E11.34	Type 2 diabetes mellitus with other retinopathy
54	E11.35	Type 2 diabetes mellitus with advanced ophthalmic disease
55	E11.36	Type 2 diabetes mellitus with diabetic cataract
56	E11.39	Type 2 diabetes mellitus with other specified ophthalmic complication
57	E11.40	Type 2 diabetes mellitus with unspecified neuropathy
58	E11.41	Type 2 diabetes mellitus with mononeuropathy
59	E11.42	Type 2 diabetes mellitus with polyneuropathy
60	E11.43	Type 2 diabetes mellitus with autonomic neuropathy
61	E11.49	Type 2 diabetes mellitus with specified neurologic complications
62	E11.51	Type 2 diabetes mellitus with peripheral angiopathy, without gangrene
63	E11.52	Type 2 diabetes mellitus with peripheral angiopathy with gangrene
64	E11.53	Type 2 diabetes mellitus with ischemic diabetic cardiopathy
65	E11.61	Type 2 diabetes mellitus with musculoskeletal and connective tissue complication
66	E11.62	Type 2 diabetes mellitus with skin and subcutaneous tissue complication
67	E11.63	Type 2 diabetes mellitus with periodontal complication
68	E11.64	Type 2 diabetes mellitus with hypoglycaemia
69	E11.65	Type 2 diabetes mellitus with poor control
70	E11.69	Type 2 diabetes mellitus with other specified complication
71	E11.71	Type 2 diabetes mellitus with multiple microvascular complications
72	E11.72	Type 2 diabetes mellitus with resistance to insulin
73	E11.73	Type 2 diabetes mellitus with foot ulcer, multiple causes
74	E11.8	Type 2 diabetes mellitus with unspecified complications
75	E11.9	Type 2 diabetes mellitus without complications
76	E13.01	Other specified diabetes mellitus with hyperosmolarity, without noncetotic hypergicemic - hyperosmotic coma
77	E13.02	Other specified diabetes mellitus with hyperosmolarity,with coma
78	E13.11	Other specified diabetes mellitus with ketoacidosis, without coma
79	E13.12	Other specified diabetes mellitus with ketoacidosis, with coma
80	E13.13	Other specified diabetes mellitus with lactic acidosis, without coma
81	E13.14	Other specified diabetes mellitus with lactic acidosis, with coma
82	E13.15	Other specified diabetes mellitus with ketoacidosis, with lactic acidosis, without coma
83	E13.16	Other specified diabetes mellitus with ketoacidosis, with lactic acidosis, with coma
84	E13.21	Other specified diabetes mellitus with incipient diabetic nephropathy
85	E13.22	Other specified diabetes mellitus with established or advanced kidney disease
86	E13.23	Other specified diabetes mellitus with end stage renal disease
87	E13.29	Other specified diabetes mellitus with other specified kidney complication

88	E13.31	Other specified diabetes mellitus with background retinopathy
89	E13.32	Other specified diabetes mellitus with preproliferative retinopathy
90	E13.33	Other specified diabetes mellitus with proliferative retinopathy
91	E13.34	Other specified diabetes mellitus with other retinopathy
92	E13.35	Other specified diabetes mellitus with advanced ophthalmic disease
93	E13.36	Other specified diabetes mellitus with diabetic cataract
94	E13.39	Other specified diabetes mellitus with other specified ophthalmic complication not elsewhere classified
95	E13.40	Other specified diabetes mellitus with diabetic neuropathy, unspecified
96	E13.41	Other specified diabetes mellitus with mononeuropathy
97	E13.42	Other specified diabetes mellitus with polyneuropathy
98	E13.43	Other specified diabetes mellitus with autonomic neuropathy
99	E13.49	Other specified diabetes mellitus with other diabetic neurological complication
100	E13.51	Other specified diabetes mellitus with peripheral angiopathy without gangrene
101	E13.52	Other specified diabetes mellitus with peripheral angiopathy with gangrene
102	E13.53	Other specified diabetes mellitus with diabetic ischemic cardiomiopathy
103	E13.61	Other specified diabetes mellitus with musculoskeletal and connective tissue complication
104	E13.62	Other specified diabetes mellitus with skin and subcutaneous tissue complication
105	E13.63	Other specified diabetes mellitus with periodontal complication
106	E13.64	Other specified diabetes mellitus with hypoglycaemia
107	E13.65	Other specified diabetes mellitus with poor control, so described
108	E13.69	Other specified diabetes mellitus with other specified complication, not elsewhere classified
109	E13.71	Other specified diabetes mellitus with multiple microvascular complications
110	E13.72	Other specified diabetes mellitus with insuline resistency
111	E13.73	Other specified diabetes mellitus with foot ulcer due to multiple causes
112	E13.8	Other specified diabetes mellitus with unspecified complications
113	E13.9	Other specified diabetes mellitus without complications
114	E14.01	Unspecified diabetes mellitus with
115	E14.02	Unspecified diabetes mellitus with with hiperosmolarity and coma
116	E14.11	Unspecified diabetes mellitus with ketoacidosis without coma
117	E14.12	Unspecified diabetes mellitus with ketoacidosis with coma
118	E14.13	Unspecified diabetes mellitus with lactic acidosis without coma
119	E14.14	Unspecified diabetes mellitus with lactic acidosis with coma
120	E14.15	Unspecified diabetes mellitus with ketoacidosis with lactic acidosis without coma
121	E14.16	Unspecified diabetes mellitus with ketoacidosis with lactic acidosis with coma
122	E14.21	Unspecified diabetes mellitus with incipient diabetic nephropathy
123	E14.22	Unspecified diabetes mellitus with established kidney disease
124	E14.23	Unspecified diabetes mellitus with end stage kidney disease
125	E14.29	Unspecified diabetes mellitus with other specified kidney complication not elsewhere classified
126	E14.31	Unspecified diabetes mellitus with background retinopathy

127	E14.32	Unspecified diabetes mellitus with preproliferative retinopathy
128	E14.33	Unspecified diabetes mellitus with proliferative retinopathy
129	E14.34	Unspecified diabetes mellitus with other retinopathy
130	E14.35	Unspecified diabetes mellitus with advanced ophthalmic disease
131	E14.36	Unspecified diabetes mellitus with diabetic cataracta
132	E14.39	Unspecified diabetes mellitus with other specified ophthalmic complication not elsewhere classified
133	E14.40	Unspecified diabetes mellitus with unspecified neuropathy
134	E14.41	Unspecified diabetes mellitus with mononeuropathy
135	E14.42	Unspecified diabetes mellitus with polyneuropathy
136	E14.43	Unspecified diabetes mellitus with autonomic neuropathy
137	E14.49	Unspecified diabetes mellitus with other specified neurologic complications
138	E14.51	Unspecified diabetes mellitus with peripheral angiopathy without gangrene
139	E14.52	Unspecified diabetes mellitus with peripheral angiopathy with gangrene
140	E14.53	Unspecified diabetes mellitus with diabetic ischemic cardiomiopathy
141	E14.61	Unspecified diabetes mellitus with musculoskeletal and connective tissue complication
142	E14.62	Unspecified diabetes mellitus with skin and subcutaneous tissue complication
143	E14.63	Unspecified diabetes mellitus with periodontal complication
144	E14.64	Unspecified diabetes mellitus with hypoglycaemia
145	E14.65	Unspecified diabetes mellitus with poor control, so described
146	E14.69	Unspecified diabetes mellitus with other specified complication, not elsewhere classified
147	E14.71	Unspecified diabetes mellitus with multiple microvascular complications
148	E14.72	Unspecified diabetes mellitus with insulin resistency
149	E14.73	Unspecified diabetes mellitus with foot ulcer due to multiple causes
150	E14.8	Unspecified diabetes mellitus with unspecified complications
151	E14.9	Unspecified diabetes mellitus without complications
152	E24.4	Alcohol-induced pseudo-Cushing's syndrome
153	E66.0	Obesity due to excess calories
154	E66.1	Drug-induced obesity
155	E66.2	Extreme obesity with alveolar hypoventilation
156	E66.8	Other obesity
157	E66.9	Obesity, unspecified
158	E67.8	Other specified hyperalimentation
159	E75.5	Other lipid storage disorders
160	E75.6	Lipid storage disorder, unspecified
161	E78.0	Pure hypercholesterolaemia
162	E78.2	Mixed hyperlipidaemia
163	E78.4	Other hyperlipidaemia
164	E78.5	Hyperlipidaemia, unspecified
165	F10.0	Mental and behavioural disorders due to use of alcohol, acute intoxication

166	F10.1	Mental and behavioural disorders due to use of alcohol, harmful use
167	F10.2	Mental and behavioural disorders due to use of alcohol, dependence syndrome
168	F10.3	Mental and behavioural disorders due to use of alcohol, withdrawal state
169	F10.4	Mental and behavioural disorders due to use of alcohol, withdrawal state with delirium
170	F10.5	Mental and behavioural disorders due to use of alcohol, psychotic disorder
171	F10.6	Mental and behavioural disorders due to use of alcohol, amnesic syndrome
172	F10.7	Mental and behavioural disorders due to use of alcohol, residual and late-onset psychotic disorder
173	F10.8	Mental and behavioural disorders due to use of alcohol, other mental and behavioural disorders
174	F10.9	Mental and behavioural disorders due to use of alcohol, unspecified mental and behavioural disorder
175	F17.0	Mental and behavioural disorders due to use of tobacco, acute intoxication
176	F17.1	Mental and behavioural disorders due to use of tobacco, harmful use
177	F17.2	Mental and behavioural disorders due to use of tobacco, dependence syndrome
178	F17.3	Mental and behavioural disorders due to use of tobacco, withdrawal state
179	F17.4	Mental and behavioural disorders due to use of tobacco, withdrawal state with delirium
180	F17.5	Mental and behavioural disorders due to use of tobacco, psychotic disorder
181	F17.6	Mental and behavioural disorders due to use of tobacco, amnesic syndrome
182	F17.7	Mental and behavioural disorders due to use of tobacco, residual and late-onset psychotic disorder
183	F17.8	Mental and behavioural disorders due to use of tobacco, other mental and behavioural disorders
184	F17.9	Mental and behavioural disorders due to use of tobacco, unspecified mental and behavioural disorder
185	F50.4	Overeating associated with other psychological disturbances
186	G31.2	Degeneration of nervous system due to alcohol
187	G45.1	Carotid artery syndrome (hemispheric)
188	G45.2	Multiple and bilateral precerebral artery syndromes
189	G45.8	Other transient cerebral ischaemic attacks and related syndromes
190	G45.9	Transient cerebral ischaemic attack, unspecified
191	G46.3*	Brain stem stroke syndrome (I60-I67+)
192	G46.8*	Other vascular syndromes of brain in cerebrovascular diseases (I60-I67+)
193	G62.1	Alcoholic polyneuropathy
194	G72.1	Alcoholic myopathy
195	I10	Essential (primary) hypertension
196	I11.0	Hypertensive heart disease with (congestive) heart failure
197	I11.9	Hypertensive heart disease without (congestive) heart failure
198	I12.0	Hypertensive renal disease with renal failure
199	I12.9	Hypertensive renal disease without renal failure
200	I13.0	Hypertensive heart and renal disease with (congestive) heart failure
201	I13.1	Hypertensive heart and renal disease with renal failure

202	I13.2	Hypertensive heart and renal disease with both (congestive) heart failure and renal failure
203	I13.9	Hypertensive heart and renal disease, unspecified
204	I15.0	Renovascular hypertension
205	I15.1	Hypertension secondary to other renal disorders
206	I15.2	Hypertension secondary to endocrine disorders
207	I15.8	Other secondary hypertension
208	I15.9	Secondary hypertension, unspecified
209	I24.0	Coronary thrombosis not resulting in myocardial infarction
210	I25.11	Atherosclerotic heart disease of native coronary artery
211	I25.4	Coronary artery aneurysm
212	I42.6	Alcoholic cardiomyopathy
213	I63.0	Cerebral infarction due to thrombosis of precerebral arteries
214	I63.1	Cerebral infarction due to embolism of precerebral arteries
215	I63.2	Cerebral infarction due to unspecified occlusion or stenosis of precerebral arteries
216	I63.3	Cerebral infarction due to thrombosis of cerebral arteries
217	I63.4	Cerebral infarction due to embolism of cerebral arteries
218	I63.5	Cerebral infarction due to unspecified occlusion or stenosis of cerebral arteries
219	I63.6	Cerebral infarction due to cerebral venous thrombosis, nonpyogenic
220	I63.8	Other cerebral infarction
221	I63.9	Cerebral infarction, unspecified
222	I64	Stroke, not specified as haemorrhage or infarction
223	I65.0	Occlusion and stenosis of vertebral artery
224	I65.1	Occlusion and stenosis of basilar artery
225	I65.2	Occlusion and stenosis of carotid artery
226	I65.3	Occlusion and stenosis of multiple and bilateral precerebral arteries
227	I65.8	Occlusion and stenosis of other precerebral artery
228	I65.9	Occlusion and stenosis of unspecified precerebral artery
229	I66.0	Occlusion and stenosis of middle cerebral artery
230	I66.1	Occlusion and stenosis of anterior cerebral artery
231	I66.2	Occlusion and stenosis of posterior cerebral artery
232	I66.3	Occlusion and stenosis of cerebellar arteries
233	I66.4	Occlusion and stenosis of multiple and bilateral cerebral arteries
234	I66.8	Occlusion and stenosis of other cerebral artery
235	I66.9	Occlusion and stenosis of unspecified cerebral artery
236	I67.4	Hypertensive encephalopathy
237	I67.8	Other specified cerebrovascular diseases
238	I67.9	Cerebrovascular disease, unspecified
239	I68.8*	Other cerebrovascular disorders in diseases classified elsewhere
240	I69.3	Sequelae of cerebral infarction
241	I69.4	Sequelae of stroke, not specified as haemorrhage or infarction

242	I69.8	Sequelae of other and unspecified cerebrovascular diseases
243	I70.0	Atherosclerosis of aorta
244	I70.20	Atherosclerosis of arteries of extremities without gangrene
245	I70.21	Atherosclerosis of arteries of extremities with gangrene
246	I70.22	Atherosclerosis of native arteries of extremities with rest pain
247	I70.23	Atherosclerosis of native arteries of right leg with ulceration
248	I70.24	Atherosclerosis of arteries of extremities with gangrene
249	I70.8	Atherosclerosis of other arteries
250	I70.9	Generalized and unspecified atherosclerosis
251	I73.0	Raynaud's syndrome
252	I73.1	Thromboangiitis obliterans [Buerger]
253	I73.8	Other specified peripheral vascular diseases
254	I73.9	Peripheral vascular disease, unspecified
255	I74.0	Embolism and thrombosis of abdominal aorta
256	I74.1	Embolism and thrombosis of other and unspecified parts of aorta
257	I74.2	Embolism and thrombosis of arteries of upper extremities
258	I74.3	Embolism and thrombosis of arteries of lower extremities
259	I74.4	Embolism and thrombosis of arteries of extremities, unspecified
260	I74.5	Embolism and thrombosis of Iliac artery
261	I74.8	Embolism and thrombosis of other arteries
262	I74.9	Embolism and thrombosis of unspecified artery
263	I77.1	Stricture of artery
264	I77.5	Necrosis of artery
265	I77.6	Arteritis, unspecified
266	I77.8	Other specified disorders of arteries and arterioles
267	I77.9	Disorder of arteries and arterioles, unspecified
268	I78.0	Hereditary haemorrhagic telangiectasia
269	I78.8	Other diseases of capillaries
270	I78.9	Disease of capillaries, unspecified
271	I79.1*	Aortitis in diseases classified elsewhere
272	I79.2*	Peripheral angiopathy in diseases classified elsewhere
273	I79.8*	Other disorders of arteries, arterioles and capillaries in diseases classified elsewhere
274	I98.8*	Other specified disorders of circulatory system in diseases classified elsewhere
275	I99	Other and unspecified disorders of circulatory system
276	K29.2	Alcoholic gastritis
277	K70.0	Alcoholic fatty liver
278	K70.1	Alcoholic hepatitis
279	K70.2	Alcoholic fibrosis and sclerosis of liver
280	K70.3	Alcoholic cirrhosis of liver
281	K70.4	Alcoholic hepatic failure

282	K70.9	Alcoholic liver disease, unspecified
283	K86.0	Alcohol-induced chronic pancreatitis
284	O10.0	Pre-existing essential hypertension complicating pregnancy, childbirth and the puerperium
285	O10.4	Pre-existing secondary hypertension complicating pregnancy, childbirth and the puerperium
286	O10.9	Unspecified pre-existing hypertension complicating pregnancy, childbirth and the puerperium
287	O13	Gestational [pregnancy-induced] hypertension
288	O16	Unspecified maternal hypertension
289	O24.0	Pre-existing type 1 diabetes mellitus
290	O24.11	Pre-existing type 2 diabetes mellitus, in pregnancy, without insulin
291	O24.12	Pre-existing type 2 diabetes mellitus, in pregnancy, with insulin
292	O24.21	Pre-existing diabetes mellitus in pregnancy, otherwise specified, without insuline
293	O24.22	Pre-existing diabetes mellitus in pregnancy, otherwise specified, withinsuline
294	O24.31	Unspecified pre-existing diabetes mellitus in pregnancy, without insulin
295	O24.32	Unspecified pre-existing diabetes mellitus in pregnancy, with insulin
296	O24.41	Gestational diabetes mellitus in pregnancy, without insulin
297	O24.42	Gestational diabetes mellitus in pregnancy, with insulin
298	O24.91	Unspecified diabetes mellitus in pregnancy, without insulin
299	O24.92	Unspecified diabetes mellitus in pregnancy, with insulin
300	O26.0	Excessive weight gain in pregnancy
301	O36.6	Maternal care for excessive fetal growth
302	Q27.3	Peripheral arteriovenous malformation
303	Q27.8	Other specified congenital malformations of peripheral vascular system
304	Q27.9	Congenital malformation of peripheral vascular system, unspecified
305	R03.0	Elevated blood-pressure reading, without diagnosis of hypertension
306	R78.0	Finding of alcohol in blood
307	T38.3	Poisoning, insulin and oral hypoglycaemic [antidiabetic] drugs
308	T46.6	Poisoning, antihyperlipidaemic and antiarteriosclerotic drugs
309	T51.0	Toxic effect, ethanol
310	T51.9	Toxic effect, alcohol, unspecified
311	T65.2	Toxic effect, tobacco and nicotine
312	T82.2	Mechanical complication of coronary artery bypass and valve grafts
313	T87.3	Neuroma of amputation stump
314	T87.4	Infection of amputation stump
315	T87.5	Necrosis of amputation stump
316	T87.6	Other and unspecified complications of amputation stump
317	X45	Accidental poisoning by and exposure to alcohol
318	X65	Intentional self-poisoning by and exposure to alcohol
319	Y15	Poisoning by and exposure to alcohol, undetermined intent
320	Y42.3	Adverse effects in therapeutic use, insulin and oral hypoglycaemic [antidiabetic] drugs

321	Y52.6	Adverse effects in therapeutic use, antihyperlipidaemic and antiarteriosclerotic drugs
322	Y83.5	Abnormal reaction/later complication, without mention of misadventure at the time of the procedure, amputation of limb(s)
323	Y90.0	Blood alcohol level of less than 20 mg/100 ml
324	Y90.1	Blood alcohol level of 20-39 mg/100 ml
325	Y90.2	Blood alcohol level of 40-59 mg/100 ml
326	Y90.3	Blood alcohol level of 60-79 mg/100 ml
327	Y90.4	Blood alcohol level of 80-99 mg/100 ml
328	Y90.5	Blood alcohol level of 100-119 mg/100 ml
329	Y90.6	Blood alcohol level of 120-199 mg/100 ml
330	Y90.7	Blood alcohol level of 200-239 mg/100 ml
331	Y90.8	Blood alcohol level of 240 mg/100 ml or more
332	Y90.9	Presence of alcohol in blood, level not specified
333	Y91.0	Mild alcohol intoxication
334	Y91.1	Moderate alcohol intoxication
335	Y91.2	Severe alcohol intoxication
336	Y91.3	Very severe alcohol intoxication
337	Y91.9	Alcohol involvement, not otherwise specified
338	Z04.0	Blood-alcohol and blood-drug test
339	Z50.2	Alcohol rehabilitation
340	Z71.4	Alcohol abuse counselling and surveillance
341	Z71.6	Tobacco abuse counselling
342	Z72.0	Tobacco use
343	Z72.1	Alcohol use
344	Z81.1	Family history of alcohol abuse
345	Z81.2	Family history of tobacco abuse
346	Z83.3	Family history of diabetes mellitus
347	Z86.41	Personal history of drug abuse
348	Z86.43	Personal history of tobacco use
349	Z95.1	Presence of aortocoronary bypass graft
350	Z95.5	Presence of coronary angioplasty implant and graft

Table S3. Amputations numbers - Comparative data in 2019: national database and 61 pool hospitals. (source 1 and 2)

No.	Name of procedure	National data - total no. (%*) 2019 (Source 1)	61 hospitals pool - total no. (%*) 2019 (Source 2	%** of the 61 hospitals pool / National data 2019	p+
1	Amputation above the knee	4,264 (28.5)	1,942 (29.9)	45.8	0.108***
2	Amputation of toe	4,156 (27.8)	1,694 (26.1)	41.3	0.061***

3	Amputation of toe including metatarsal bone	2,776 (18.6)	1,223(18.9)	43.8	1.00
4	Transmetatarsal amputation	1246 (8.3)	545 (8.4)	45.3	0.380
5	Amputation below knee	1143 (7.7)	508 (7.8)	44.2	1.00
6	Reamputation of amputation stump	786 (5.3)	308 (4.3)	39.3	0.270***
7	Midtarsal amputation	396 (2.7)	199 (3.1)	50.3	0.135
8	Amputation at hip	142 (1.0)	60 (0.9)	42.3	0.417
9	Amputation of ankle through malleoli of tibia and fibula	29 (0.2)	12 (0.2)	41.4	1.00
**Total**		**14,938**	**6,488**	43.73	

***percent from the total on the column**
**** percent from the total on the line**
***** with Bonferroni correction**
+ p for one proportion test (H0: proportion of th amputations reported on the code on 61 pool of hospitals did not differ statistically significant from the proportion in the population national data**)**
No. - number

Table S4. Number of amputations from the national database (source 1) by DRG code, and year

No.	Name of procedure	National data 2016	National data 2017	National data 2018	National data 2019	National data 2020	National data 2021
1	Amputation above the knee	4,259	4,277	4,407	4,264	4,223	4,490
2	Amputation of toe	3,738	3,987	3,834	4,156	3,113	3,428
3	Amputation of toe including metatarsal bone	2,513	2,568	2,625	2,776	2,292	2,574
4	Transmetatarsal amputation	944	1,219	1,224	1,246	1,158	1,313
5	Amputation below knee	983	1,020	982	1,143	1,009	1,063
6	Reamputation of amputation stump	795	807	783	786	675	663
7	Midtarsal amputation	384	401	352	396	335	361
8	Amputation at hip	132	107	142	142	93	96
9	Amputation of ankle through malleoli of tibia and fibula	32	23	34	29	26	36
**Total**		**13,780**	**14,409**	**14,383**	**14,938**	**12,924**	**14,024**

Table S5. – Number of revascularizations (source 1) by DRG code per years

No.	Name of the procedure	National data 2016	National data 2017	National Data 2018	National Data 2019	National data 2020	National data 2021
1	Percutaneous transluminal balloon angioplasty with stenting, single stent	2,304	2,497	2,876	3,113	2,744	4,075
2	Percutaneous transluminal balloon angioplasty with stenting, multiple stents	876	927	1,174	1,536	1,290	2,009
3	Percutaneous transluminal balloon angioplasty	2,119	2,642	2,493	3,377	1,988	2,377
4	Embolectomy or thrombectomy of femoral artery	1,162	1,047	1,137	1,120	829	1,101
5	Embolectomy or thrombectomy of popliteal artery	875	791	884	833	594	821

6	Patch graft of artery using synthetic material	505	531	602	653	447	552
7	Femoro-popliteal bypass using synthetic material, above knee anastomosis	475	487	531	483	312	386
8	Embolectomy or thrombectomy of iliac artery	443	401	426	411	322	424
9	Embolectomy or thrombectomy of tibial artery	352	340	373	387	298	395
10	Patch graft of artery using autologous material	401	406	373	409	315	463
11	Embolectomy or thrombectomy of bypass graft of artery of extremities	164	187	217	204	160	184
12	Procurement of vein from limb for bypass or replacement graft	512	439	498	608	549	696
13	Embolectomy or thrombectomy of other artery	245	251	243	279	286	449
14	Aorto-femoro-femoral bypass using synthetic material	224	235	226	200	142	180
15	Femoro-popliteal bypass using synthetic material, below knee anastomosis, unilateral	196	183	212	192	189	201
16		1,925	1,909	2,140	1,942	1,566	1,862
**Total**		**12,778**	**13,273**	**14,405**	**15,747**	**12,031**	**16,175**
**Total open procedures**		7,454	7,195	7,842	7,698	5,982	7,691
**Total endovascular procedures**		5,324	6,078	6,563	8,049	6,049	8,484

Table S6 Revascularisation numbers - Comparative data in 2019: national database and 61 pool hospitals (source 1 and 2)

**No.**	**Name of the procedure**	**National Data - Total no. (*%) 2019 (Source 1)**	**61 hospitals pool - Total no. (*%) 2019 (Source 2)**	**% 61 hospitals pool / National data 2019**	p^{+}
1	Percutaneous transluminal balloon angioplasty with stenting, single stent	3,113 (19.8)	1,707 (25.4)	54.83	**<0.001*** **
2	Percutaneous transluminal balloon angioplasty with stenting, multiple stents	1,536 (9.8)	964 (14.4)	62.76	**<0.001*** **
3	Percutaneous transluminal balloon angioplasty	3,377 (21.4)	696 (10.4)	20.61	**<0.001*** **
4	Embolectomy or thrombectomy of femoral artery	1,120 (7.1)	536 (8)	47.86	0.064***
5	Embolectomy or thrombectomy of popliteal artery	833 (5.3)	415 (6.2)	49.82	**0.016*** **
6	Patch angioplasty of artery using synthetic material	653 (4.1)	307 (4.6)	47.01	0.624
7	Femoro-popliteal bypass using synthetic material, above knee anastomosis	483 (3.1)	226 (3.4)	46.79	0.175
8	Embolectomy or thrombectomy of iliac artery	411 (2.6)	216 (3.2)	52.55	**0.032*** **
9	Embolectomy or thrombectomy of posterior tibial artery	387 (2.5)	143 (2.1)	36.95	0.576
10	Patch angioplasty of artery using autologous material	409 (2.6)	138 (2.1)	33.74	0.16
11	Embolectomy or thrombectomy of bypass graft of artery of extremities	204 (1.3)	117 (1.7)	57.35	**0.048*** **
12	Procurement of vein from limb for bypass or replacement graft	608 (3.9)	115 (1.7)	18.91	**<0.001*** **

13	Embolectomy or thrombectomy of other artery	279 (1.8)	103 (1.5)	36.92	0.064***
14	Aorto-femoro-femoral bypass using synthetic material	200 (1.3)	102 (1.5)	51.00	0.148
15	Femoro-popliteal bypass using synthetic material, below knee anastomosis, unilateral	192 (1.2)	99 (1.5)	51.56	0.384
16		1,942 (12.3)	831 (12.4)	42.79	0.803
**Total**		**15,747**	**6,715**	**42.64**	

*percent from the total on the column
** percent from the total number of cases from National Data 2019 (Source 1)
*** with Bonferroni correction
+ p for one proportion test (H0: proportion of the vascular reported on the code on 61 pool of hospitals did not differ statistically significant from the proportion in the population national data

Table S7. – Risk factors – age distribution at people with amputations – extracted from the 61 hospital pool (source2)

Risk factor/age group	**under 30 years (n=15)**	**30-39 years (n=52)**	**40-49 years (n=302)**	**50-59 years (n=951)**	**60-69 years (n=2129)**	**70-79 years (n=1593)**	**80 and above (n=943)**	**Arithmetic mean± standard deviation %**
PAD and DM	0 (0)	14 (26.9)	97 (32.1)	412 (43.3)	1052 (49.4)	736 (46.2)	313 (33.2)	33.02± 16.72
PAD without DM	9 (60)	15 (28.8)	71 (23.5)	216 (22.7)	545 (25.6)	518 (32.5)	483 (51.2)	34.91± 14.74
PAD	9 (60)	29 (55.8)	168 (55.6)	628 (66)	1597 (75)	1254 (78.7)	796 (84.4)	67.94± 11.57
DM without PAD	0 (6.7)	4 (19.2)	8 (23.8)	47 (24.3)	110 (18.7)	77 (13.9)	38 (7.4)	16.3±7.22
DM with gangrene /ulcer	0 (0)	11 (21.2)	76 (25.2)	319 (33.5)	780 (36.6)	545 (34.2)	205 (21.7)	24.64± 12.54
DM	1 (6.7)	10 (46.2)	72 (56)	231 (67.6)	399 (68.2)	222 (60.1)	70 (40.6)	49.33± 21.43
Revascularization	1 (0)	24 (7.7)	169 (2.6)	643 (4.9)	1451 (5.2)	958 (4.8)	383 (4)	4.19±2.39
Hypertension	1 (6.7)	8 (15.4)	80 (26.5)	413 (43.4)	1210 (56.8)	941 (59.1)	546 (57.9)	37.97± 21.79
Ischemic stroke	0 (0)	3 (5.8)	3 (1)	62 (6.5)	226 (10.6)	219 (13.7)	133 (14.1)	7.39±5.7
Hyperlipidemia	0 (0)	4 (7.7)	21 (7)	76 (8)	192 (9)	148 (9.3)	49 (5.2)	6.59±3.21
Obesity	1 (6.7)	7 (13.5)	30 (9.9)	121 (12.7)	283 (13.3)	169 (10.6)	57 (6)	10.39± 3.06
Smoking	1 (6.7)	29 (55.8)	168 (55.6)	628 (66)	1597 (75)	1254 (78.7)	796 (84.4)	60.32± 26.11

Alcoholism	0 (0)	3 (5.8)	13 (4.3)	39 (4.1)	74 (3.5)	34 (2.1)	5 (0.5)	2.9±2.11

Risk factor/age group	under 30 years (n=15)	30 - 34 years (n=20)	35 - 39 years (n=32)	40 - 44 years (n=84)	45 - 49 years (n=218)	50 - 54 years (n=409)	55 - 59 years (n=542)	60 - 64 years (n=981)	65 - 69 years (n=1148)	70 - 74 years (n=903)	75 - 79 years (n=690)	80 - 84 years (n=560)	85 and above (n=383)	media	Deviatia standard
Smoking (n=404)	1	4	4	12	25	48	45	86	100	39	23	14	3	61.95	10.72
Hyperlipidemia (n=490)	0	2	2	7	14	26	50	87	105	87	61	30	19	67	10.06
Diebtes Mellitus (3629)	1	8	16	48	121	269	374	669	782	582	376	268	115	66.12	10.46
PAD (n=4481)	9	14	15	47	121	249	379	713	884	707	547	470	326	68.29	11.53
Revascularization (n=284)	0	2	2	0	8	20	27	52	58	47	30	29	9	67	10.47
DM si Gangrene /ulcer (n=1936?) 1005	0	5	6	22	54	133	186	359	421	332	213	154	51	66	10,47
PAD si Diabet (2624)	0	6	8	31	66	161	251	462	590	449	287	219	94	67.01	10.37
Hypertension (n=3199)	1	3	5	13	67	159	254	551	659	531	410	333	213	69	10.10
Ischemic Stroke (n=646	0	0	3	1	2	21	41	107	119	116	103	78	55	71	9.38
Coronary Disease (n=107)	0	0	0	1	1	8	6	24	17	19	15	13	3	68	9.6
Carotid Disease (n=90)	0	0	0	0	0	3	6	18	17	24	11	8	3	69	8.32
Obesity (n=668)	1	2	5	5	25	48	73	123	160	112	57	42	15	66	9.98
Alcoholism (n=168)	0	1	2	4	9	19	20	35	39	21	13	2	3	63	10.24

Table S8. – Discharge ward (source 2) for people with amputations in 2019

No.	Discharge clinic	No. of amputations	% of total, 95% CI
1	General Surgery	4,327	72.3 (71.2; 73.4)
2	Vascular Surgery	1,143	19.1 (18.1; 20.1)

3	Plastic and Reconstructive Suegery	161	2.7 (2.3; 3.1)
4	Cardiovascular Surgery	101	1.7 (1.4; 2)
5	Orthopedy and Traumatology	92	1.5 (1.2; 1.8)
6	Osteo-articular TBC	21	0.4 (0.2; 0.6)
7	Internal Medicine	20	0.3 (0.2; 0.4)
8	Cardiology	16	0.3 (0.2; 0.4)
9	Gynecology	15	0.3 (0.2; 0.4)
10	Neurology	10	0.2 (0.1; 0.3)
11	Acute Psichiatry	10	0.2 (0.1; 0.3)
12	Infectious Diseases	9	0.2 (0.1; 0.3)
13	Burns	9	0.2 (0.1; 0.3)
14	Dermatology	8	0.1 (0; 0.2)
15	Diabetology	7	0.1 (0; 0.2)
16	Other	106	1.8 (1.5; 2.1)
**Total**		**5,985**	**100**
**No. – number; CI – Confidence interval**			

Table S9. – Surgeon specialty realizing the amputation procedure (source 2) for people with amputations in 2019

**No.**	**Specialty of surgeon**	**No. of amputations**	**% of total, 95% CI**
1	General Surgeon	4,374	73.1 (72; 74.2)
2	Vascular Surgeon	1,042	17.4 (16.4; 18.4)
3	Plastic Surgeon	206	3.4 (2.9; 3.9)
4	Cardiovascular surgeon	119	2 (1.6; 2.4)
5	Orthopedic	96	1.6 (1.3; 1.9)
6	No medical doctor	87	1.5 (1.2; 1.8)
7	Thoracic Surgery	20	0.3 (0.2; 0.4)
8	Anesthesiology	19	0.3 (0.2; 0.4)
9	Cardiology	11	0.9 (0.7; 1.1)
10	Emergency Internal Medicine	3	0.1 (0; 0.2)
11	General Pharmacy	2	0.03 (0; 0.1)
12	Internal Medicine	2	0.03 (0; 0.1)
13	Dermatology	1	0.02 (0; 0.1)
14	Diabetologist	1	0.02 (0; 0.1)
15	Neurosurgeon	1	0.02 (0; 0.1)
16	Gynecologist	1	0.02 (0; 0.1)
**Total**		**5,985**	
**No. – number; CI – Confidence interval**			

Table S10 (Source 2) **Intra-hospital mortality in people with amputations**

No.	Age group (years)	No. of discarded cases 2019	p compare to 18-49 years age group	RR, 95% CI compare to 18-49 years age group
**1**	18 - 49 (n=369)	18 (4.9)	-	-
**2**	50 - 54 (n=409)	18 (4.4)	0.752	-
**3**	55 - 59 (n=542)	29 (5.4)	0.752	-
**4**	60 – 64 (n=981)	68 (6.9)	0.169	-
**5**	65 - 69 (n=1148)	117 (10.2)	0.002	2.09 (1.29; 3.38)
**6**	70 – 74 (n=903)	113 (12.5)	<0.001	2.57 (1.58; 4.16)
**7**	75 - 79 (n=690)	85 (12.3)	<0.001	2.53 (1.54; 4.75)
**8**	80 – 84 (n=560)	88 (15.7)	<0.001	3.22 (1.97; 5.26)
**9**	85 > (n=383)	74 (19.3)	<0.001	3.96 (2.41; 6.5)
**Total**		610 (10.2)		

NO. – NUMBER; RR – RELATIVE RISK; CI – CONFIDENCE INTERVAL
